# Cardioprotective effects of co-administration of thymoquinone and ischemic postconditioning in diabetic rats

**DOI:** 10.22038/ijbms.2021.47670.10981

**Published:** 2021-07

**Authors:** Junchuan Ran, Huanglin Xu, Wenyuan Li

**Affiliations:** 1 Department of Cardiology, Gansu Gem Flower Hospital, Lanzhou, Gansu, 730060, China; 2 Department of Cardiology, Xigu People’s Hospital,Lanzhou, Gansu, 730060, China

**Keywords:** Apoptosis, Diabetes, Inflammation, Ischemic postconditioning, Polyphenols

## Abstract

**Objective(s):**

Ischemia/reperfusion (I/R) is a leading cause of myocardial infarction (MI) injury, contributing to excess injury to cardiac tissues involved in inflammation, apoptosis, and oxidative stress. The present study was conducted to examine the effects of combined thymoquinone (TQ) with ischemic postconditioning (IPostC) therapy on apoptosis and inflammation due to I/R injury in diabetic rat hearts.

**Materials and Methods:**

A single dose injection of streptozotocin (STZ; 60 mg/kg) was administered to thirty-two Wistar male rats to induce diabetes. Hearts were fixed on a Langendorff setting and exposed to a 30 min regional ischemia subsequently to 60 min reperfusion. IPostC was induced at the onset of reperfusion by 3 cycles of 30 sec R/I. ELISA, Western blotting assay, and TUNEL staining were applied to assess the cardioprotective effect of IPostC and TQ against I/R injury in diabetic and non-diabetic rats.

**Results:**

Administration of TQ alone in non-diabetic isolated hearts significantly diminished CK-MB, TNF-α, IL-1β, and apoptosis and enhanced p-GSK-3β and Bcl-2 (*P*<0.05). Following administration of TQ, the cardioprotective effects of IPostC by elevating p-GSK-3β and Bcl-2 and alleviating apoptosis and inflammation were reestablished compared with non-IPostC diabetic hearts.

**Conclusion:**

These results provide substantial evidence that co-administration of TQ plus IPostC can exert cardioprotective effects on diabetic myocardium during I/R damage by attenuating the inflammatory response and apoptosis.

## Introduction

The incidence of cardiovascular disorder in diabetics is considerably more prominent than in the non-diabetic population. In this regard, ischemic heart disease (IHD) is the most prevalent leading factor of disability and fatalities in diabetic patients worldwide (1). The recovery of blood supply is the most essential therapy in IHD. At present, timely reperfusion is a major therapeutic strategy to treat myocardial ischemia, however, reperfusion itself can result in lethal cardiac damage (2). The prominent clinical manifestations of this damage include left ventricular extracellular matrix remodeling, persistent ventricular systolic dysfunction, ventricular arrhythmias, irreversible cardiomyocyte injury, microvascular dysfunction, progressive cell death, and finally, heart failure (3). Furthermore, oxidative stress and cell apoptosis describe pivotal factors in the occurrence and extension of myocardial ischemia/reperfusion (I/R) injury. Therefore, it is important to further investigate the mechanism underlying I/R injury from various perspectives and how to effectively minimize this damage through intervention to protect the heart tissue (4).

Ischemic postconditioning (IPostC) can be defined as a manner of cardioprotection targeting of I/R injury, which is conducted after reopening of the occluded artery and before final restoration (5). Indeed, IPostC stimulates the endogenous mechanisms that decrease the multiple interactions of reperfusion damage (6). In this regard, the inflammatory response has a pivotal role in I/R damage. Inflammatory signaling pathways produced during reperfusion damage activate NF-κB and lead to up-regulation of a broad range of crucial pro-inflammatory cytokines, such as tumor necrosis factor-alpha (TNF-α) and interleukin-1 (IL-1), IL-6, IL-8, IL-12 which result in initiating inflammatory responses in the heart. In diabetic patients, the inflammatory response to I/R injury (IRI) is intensified because of the stimulatory role of diabetes in the activation of adhesion molecules and cytokines and accumulation of leukocytes (7). 

Besides, mitochondria play a pivotal role in cardioprotection (8). Damage to the outer membrane of mitochondria (OMOM) is concomitant with the actuation of the B-cell lymphoma-2 (Bcl-2) which contributes to permeabilization, caspase activation, and cell apoptosis. Also, immense oxidative stress can contribute to an abruptly enhanced inner mitochondrial membrane permeability due to the opening of mitochondrial permeability transition pore (mPTP) (9). IPostC inhibits mPTP through overexpression of phosphoinositide-3-kinase/protein kinase B (PI3K/PKB) pathway and whereby inactivation of glycogen synthase kinase-3 beta (GSK-3β) in the myocardium. Activation of the PI3K/Akt/GSK-3β pathway effectively alleviates cardiac cell apoptosis and IRI (10). Studies revealed that chronic diabetes predispose the cardiac cells to IRI. In a sense, IPostC alone could not exert considerable cardioprotective effects on diabetic myocardium from reperfusion (11).

Recently, natural products have drawn extensive attention due to their high efficiency and low side-effects, which is a rational mechanism in augmenting the reaction of cells to endure I/R injury (12). Among these, thymoquinone (TQ) is the crucial component of the essential oil obtained from *Nigella sativa L.* seeds (black cumin) (13). TQ is an active quinone derivative, whose beneficial therapeutic effects are due to its anti-ischemic, anti myocardial and perivascular fibrosis, anti-hypertensive, anti-hypercholesterolemia, antihistamine, anti-apoptotic, hypolipidemic, hypoglycemic, anti-inflammatory, anti-cancer, and immunity-boosting effects (14-18). Recent studies have demonstrated that TQ could reduce I/R injury in several organs such as the brain, liver, spinal cord, heart, and renal tissue (19, 20). Bearing all this in mind, the underlying molecular mechanism of TQ in the heart tissue is still under investigation.

Indeed, the risk of ischemic conditions including myocardial infarction (MI) increases in diabetic patients and they may mount an exaggerated response to I/R. Hence, the present study was designed to first examine whether TQ and IPostC ameliorate myocardium IRI in type 1 diabetic state and secondly illuminate the cardioprotective roles of TQ and IPostC in inhibiting apoptosis and inflammation mediators in these pathways. 

## Materials and Methods

***Experimental preparation***

Fifty-six twelve-weeks-old Wistar male rats (weighing 260±40 g) were supplied from the animal center facility of the research center and maintained under standard animal room conditions [constant 12 hr light/dark cycle, temperature (25±2 °C), and relative humidity (60±5%)]. For the present study, experimental procedures were conducted according to the National Institutes of Health (NIH Publication No. 85-23, revised in 1996) Guidelines for the Care and Use of Laboratory Animals. Initially, animals were randomly separated into two main classifications of diabetic and control (non-diabetic) groups.

***Induction of diabetes***

After overnight fasting, animals received a single dose intraperitoneal (IP) injection of streptozotocin (STZ, Sigma-Aldrich, MO, USA, 60 mg/kg, IP, dissolved in 0.1 mol/l cold citrate buffer, pH 4.5). Diabetes was confirmed 72 hr after STZ injection using a glucometer and the blood samples acquired from scratching rat tails. Rats with fasting plasma glucose levels of lower than 250 mg/dl (13.8 mmol/l) were excluded from the experiment, whilst more than 13.8 mmol/l was considered diabetic. Eight weeks post-STZ diabetes induction, all animals were sacrificed and experiments were conducted on isolated perfused beating hearts in a Langendorff apparatus.

***Induction of regional I/R and IPostC on Langendorff setting***

56 rats were heparinized (500 IU/kg, IP) and anesthetized intraperitoneally with chloral hydrate (0.9 ml/100 g, IP). After median sternotomy, the hearts were rapidly isolated and immersed in Krebs–Henseleit solution (K–H). Subsequently, the hearts were cannulated through the aorta and perfused with K–H solution. A mixture of 5 % CO_2_ and 95 % O_2_ was bubbled via the perfusate, and pH was kept between 7.35 and 7.45. The hearts were perfused at a constant mean pressure of 75 mmHg throughout the investigation. The thermostatically controlled water circulator (Satchwell Sunvic, UK) maintained the perfusate and bath temperatures at 37 °C. After a 20 min stabilization period, the left anterior descending (LAD) coronary artery was ligated by passing a 5-0 silk suture placed around LAD close to its origin. Exposure to 30 min of ischemia induced by disruption of the aortic supply and 60 min of full reperfusion followed. A critical drop (down to 30–40 % of its baseline value) in coronary flow at the onset of index ischemia and the consequent recovery of the coronary flow in the reperfusion period showed success in inducing the I/R cycle. IPostC was performed by three intermittent cycles of 30 sec R/I (3 cycles of 30 sec).

***Experimental design***

The experiments were conducted as follows (n=56; 7/per group); the hearts with weak contractions or with arrhythmias were excluded from the experiment and replaced with another one. The exclusion (and replacement) rate for groups was as follows: (I) Control group (C) ─ non-diabetic rats with no TQ and IPostC treatment=0; (II) Control with TQ group (C+TQ) ─ non-diabetic rats receiving TQ= 1; (III) Control with IPostC (C+IPostC) ─ non-diabetic rats with IPostC=0; (IV) Control with IPostC plus TQ (C+IPostC+TQ) ─ non-diabetic rats with IPostC plus TQ; (V) Diabetic group (D) ─ diabetic rats with no TQ and IPostC= 2; (VI) Diabetic with IPostC group (D+IPostC) ─ diabetic rats with IPostC=0; (VII) Diabetic with TQ group (D+TQ) ─ diabetic rats receiving TQ=0; (VIII) Diabetic with IPostC plus TQ group (D+IPostC+TQ) ─ diabetic rats with IPostC plus TQ=1. The arrhythmias and weak contraction or may be related to the failure in surgical procedure. In TQ-receiving groups, 5 min before the onset of reperfusion up to 10 min after reperfusion, the hearts were perfused with a K–H solution containing 50 µM TQ. 

***Assessment of cardiac enzyme activity***

The coronary effluent was collected 10 min after the beginning of reperfusion. Ischemic injury was measured using a creatine kinase (CK-MB) assay kit. The CK-MB activity was determined spectrophotometrically by using commercial kits bought from Roche Diagnostic (Mannheim, Germany). Finally, total CK-MB was presented as the average in Unit/l.

***Western blot***

Protein concentration was determined by the Bradford technique. 40 μg protein samples were electrophoresed and then transferred to polyvinylidene fluoride (PVDF, Millipore, USA) membranes. Next, 5% skim milk dissolved in Tris-buffered saline containing 0.1% Tween (TBST) was applied to block the membranes for 2 hr at room temperature. The membranes were incubated for 24 hr with primary antibodies against GSK3β (1:500, Cell Signaling Technology, USA), phosphor (Ser 9) GSK-3β (1:2000, Cell Signaling Technology), Bcl-2 (1:1500, Cell Signaling Technology), and β-actin (1:4000, Cell Signaling Technology). Upon extensive washing, the blots were probed with a goat anti-mouse horseradish peroxidase (HRP) conjugated secondary antibody (1:2500, Cell Signaling) for 2 hr at room temperature. Then, membranes were visualized by an ECL chemiluminescent system (Bio-Rad Laboratories, Hercules, California).

***Preparation of tissue homogenates***

The ischemic zone was separated and immediately frozen in liquid nitrogen and stored at -80 °C. About 0.5 g of ventricular tissue was cut into pieces in about 5 ml of ice-cold lysis buffer containing: 1.0 KH_2_PO_4 _mM/ml, 1.0 KCL mM/ml, 1.0 EDTA, 50 Tris–HCl pH 7.4, 1.0 NaF mM/ml, 1.0 Na_3_VO_4 _mM/ml, and 1% Triton 100X and protease inhibitor cocktail (Sigma-Aldrich, USA) and then homogenized with a Polytron PT-10/ST homogenizer. The homogenate was undergoing centrifugation at 12,000 ×g for 15 min at 4 °C. The Bradford technique was applied for the revealing of the concentration of proteins levels and cytokine activity in supernatants.

***Assessment of IL-1β and TNF-α levels***

The levels of serum TNF-α and IL-1β were estimated using enzyme-linked immunosorbent assay (ELISA) rat specific kit following the instructions of the kit manufacturer (eBioscience, Austria).

***Hematoxylin and eosin (H&E) staining***

Myocardial apoptosis was monitored by H&E staining. LV sections with 5 μm thickness were obtained using a microtome. The sections were then stained with H&E (Beijing Solarbio Science & Technology Co., China) and scrutinized under a light microscope (Leica Microsystems, Germany).

***Statistical analysis***

All values were assayed in triplicate independent repeated tests and expressed as mean ± standard error of the mean (SEM). A comparison between the two groups was performed by independent Student’s t-test. Comparisons of parameters between different groups were conducted by one-way analysis of variance (ANOVA) followed by Tukey-Kramer *post-hoc* test. Two-way ANOVA was applied for the comparison of parameters within groups. A value of *P*<0.05 was considered statistically significant. 

## Results

***Basic characteristics of the animals***

The general and basic characteristic data of diabetic and non-diabetic (control) rats after eight weeks of diabetes induction are indicated in Table 1. Independent t-test revealed dramatic hyperglycemia (F_ (1, 58)_=31.65, *P*<0.001) as well as diminished bodyweight (F_(1, 58)_=209, *P*<0.001) and enhanced ratio of heart weight to body weight (F _(1, 58)_ =0.51, *P*<0.001) in comparison with those of control rats.

***The activity of CK in control and diabetic rats***

**Figure 1:**
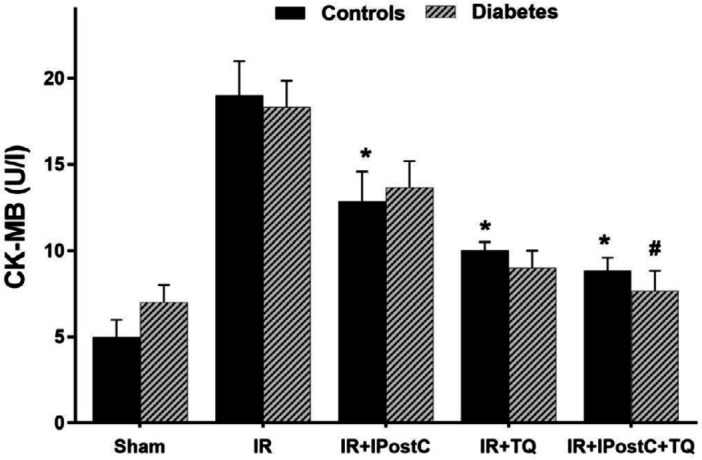
CK-MB release level (U/l) in experimental I/R hearts. *P<0.05 in comparison with the control (non-diabetic) I/R group; #*P*<0.05 in comparison with the diabetic I/R group. Mean±SEM. n=7/ group. TQ: thymoquinone; I/R: ischemic/reperfused; IPostC: ischemic postconditioning

CK-MB release level (Unit/l) in experimental I/R hearts has been displayed in Figure 1. The two-way ANOVA indicated dramatic therapeutic effect on myocardial CK-MB levels (F_(3, 60)_=8.54, *P*<0.001). The main effect of diabetes on CK-MB levels was insignificant (F_(2, 68)_=1.05, *P*=0.18), whilst the interaction effect of treatment × diabetes was dramatically significant (F_(3, 50)_=3.92, *P*=0.021). There was an insignificant difference in CK-MB levels between control and diabetic groups, as revealed by one-way ANOVA and Tukey *post hoc*. Use of IPostC at the beginning of reperfusion in the control group markedly decreased myocardial CK-MB release vs control I/R group (13.4±1.95 vs 18.1±2.4, *P*=0.03). Treatment with TQ at the end of ischemia had a similar to IPostC outcome (10.1 ±0.9, *P*=0.02). Besides, administration of TQ plus IPostC enhanced CK-MB reduction even to a greater extent (8.7±1.1 vs 18.1±2.4, *P*=0.01). In the diabetic sham group, only the effect of combination therapy (TQ plus IPostC) was significant compared with the diabetic I/R group (76±1.1 vs 17.9±2.3, *P*=0.01). 

***Inflammatory cytokines assays***

**Figure 2:**
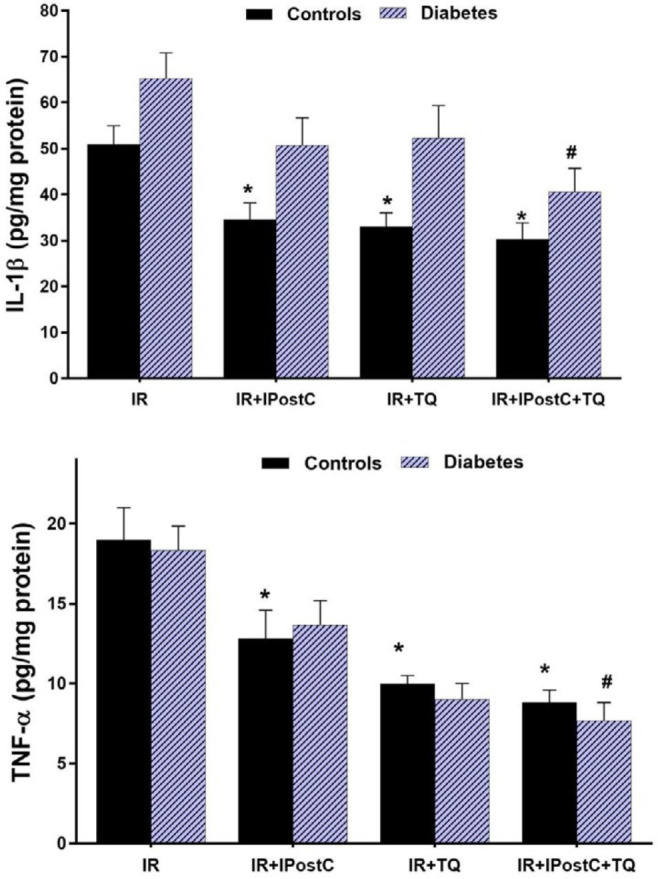
(A) IL-1β, and (B) TNF-α levels (in pg/mg of sample protein) in experimental I/R hearts. *P<0.05 in comparison with the control I/R group; **P<0.01 in comparison with the control I/R group; #*P*<0.05 in comparison with the diabetic I/R group; ##*P*<0.01 in comparison with the diabetic I/R group. Mean±SEM. n=7

TQ: thymoquinone; I/R: ischemic/reperfused; IPostC: ischemic postconditioning

The main effects of diabetes for IL-1β were dramatic (F_ (2, 48)_=28.08, *P*<0.01; F_(2, 48)_=18.91, *P*<0.01, respectively). The significant main treatments effects were found for IL-1β (F_(4, 55)_=25.19, *P*<0.01; and F_(4, 68)_=10.03, *P=*0.03, respectively). One-way ANOVA demonstrated that administration of TQ in the control I/R group markedly alleviated the levels of IL-1β, pg/mg (32.41±4.49, *P*=0.03 by TQ vs 50.8±5.48), as compared with untreated control I/R hearts. Nevertheless, in diabetic groups, the positive effects of IPostC (*P=*0.41) or TQ (*P=*0.07) were not detected (Figure 2A). Moreover, the combination of TQ and IPostC, both in healthy and diabetic hearts, dramatically reduced IL-1β levels in treated hearts in comparison with untreated I/R hearts and this effect was robust than every alone therapy along within both diabetic and control groups. IL-1β levels were 28.5±2.95 vs 50.8±5.76 pg/mg in control I/R hearts, *P=*0.01 and 40.5±5.67 vs 64.42±7.43 pg/mg in diabetic I/R hearts, *P=*0.03. Also, the main effects of diabetes and co-treatments for TNF-α were statically significant (F_(2, 48)_=11.59, *P=*0.02 and F_(4, 55)_=4.21, *P=*0.04, respectively). One-way ANOVA demonstrated that in control hearts, similar to the special effects of combination therapy (*P=*0.03), treatment with TQ alone (*P=*0.03) could significantly decrease the myocardial TNF-α levels in comparison with the control I/R group. In diabetic groups, application of TQ alone (9.6±1.5 pg/mg of sample protein, *P=*0.03) or in combination with IPostC (7.8±0.93 pg/mg of sample protein, *P=*0.01) diminished TNF-α levels as compared with untreated diabetic I/R groups (17.6±2.9 pg/mg) and co-treatment had an effect on reduction of TNF-α levels (Figure 2B).

***Phosphorylation (at Ser 9) of myocardial GSK3β***

**Figure 3:**
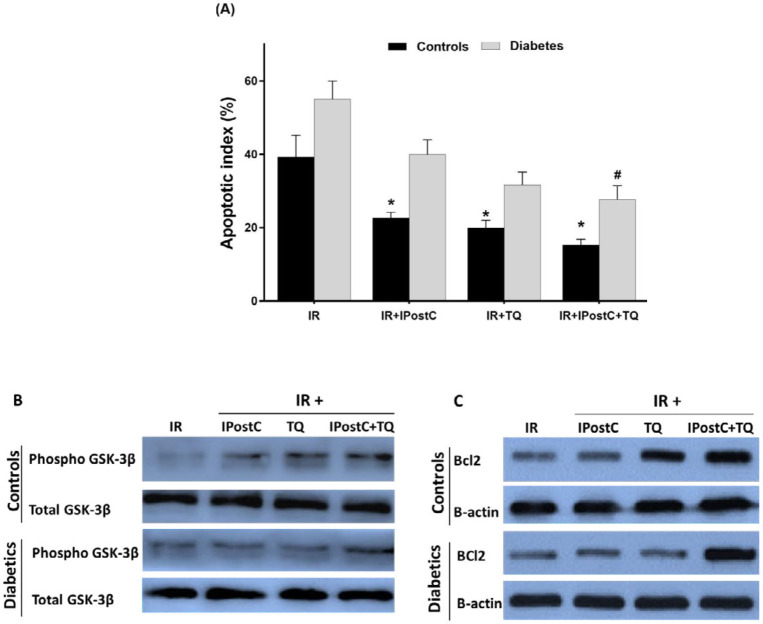
(A) Apoptotic index, (B) Phosphorylation of myocardial GSK-3β, and (C) Expression of myocardial Bcl-2 protein in experimental I/R hearts. *P<0.05 in comparison with the control I/R group; #*P*<0.05 in comparison with the diabetic I/R group. Mean±SEM. n=7

Bcl-2: B-cell lymphoma 2; I/R: ischemic/reperfused; TQ: thymoquinone; IPostC: ischemic postconditioning; GSK-3β: Glycogen synthase kinase 3 beta

In the present study, total and Ser 9-phosphorylated forms of GSK-3β, Bcl-2, and β-actin were detected using the Western blotting assay (Figures 3B, C). The total forms of GSK-3β in control and diabetic hearts were similar and there were insignificant differences between all groups. Two-way ANOVA revealed remarkable effects of diabetes (F_(2, 48)_=8.42, *P=*0.02), treatments (F_(4, 55)_=14.91, *P*<0.01) and a diabetes treatments interaction (F_(4, 55)_=3.63, *P=*0.05) on the levels of phosphorylated to the total form of GSK-3β. In non-diabetic groups, application of TQ (2.02±0.09, *P=*0.033) or their combination (1.5±0.16, *P=*0.02) significantly enhanced the levels of phosphorylated to the total form of GSK-3β (in arbitrary unit) in comparison with the corresponding I/R heart group (0.5±0.09). In diabetic groups, notwithstanding, the dramatic enhancement in the GSK-3β phosphorylation was recognized only under co-treatment (2.1±0.1 vs 0.77±0.08, *P=*0.02); the single effects of TQ or IPostC in diabetic hearts were not dramatic in comparison with those of diabetic I/R groups (Figure 3B).

***Bcl-2 protein expression levels***

Bcl-2 levels normalized by β-actin bands in control and diabetic hearts are demonstrated in Figure 5C. Two-way ANOVA analysis results revealed remarkable main effect of therapy (F_(4, 55_)=10.94, *P*<0.001) and marked interaction effect among treatment and diabetes on Bcl-2 expression levels (F_(4, 55)_= 6.07, *P=*0.02). The main effect of diabetes on Bcl-2 expression levels was not dramatic (F_(2, 48)_=2.31). Furthermore, in non-diabetic groups, IPostC alone (*P*<0.04) or in combination (*P=*0.003), dramatically elevated the relative expression of Bcl-2 protein in comparison with the I/R control group. In a sense, only significant enhancement in the Bcl-2 protein level was achieved after combining both IPostC and TQ protocols as compared with the diabetic group (*P=*0.032). Treatment of diabetic hearts with IPostC (*P=*0.69) or TQ (*P=*0.57) could not dramatically modify the expression levels of Bcl-2 (Figure 3C).

***Myocardial apoptosis after I/R***

**Figure 4:**
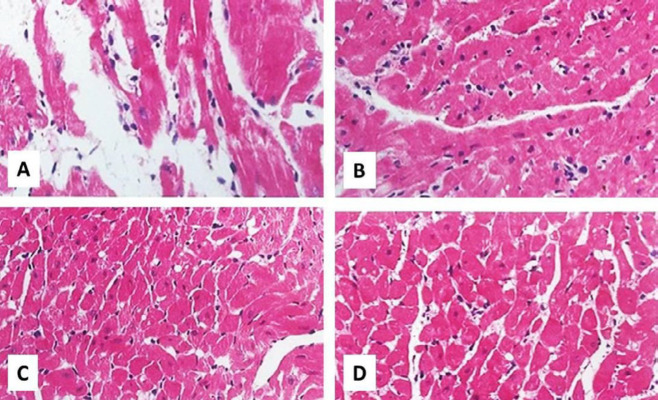
Micrographs of cardiomyocyte representing the process of apoptosis in experimental I/R hearts in control hearts. Myocardial apoptosis was identified via TUNEL staining

**Figure 5:**
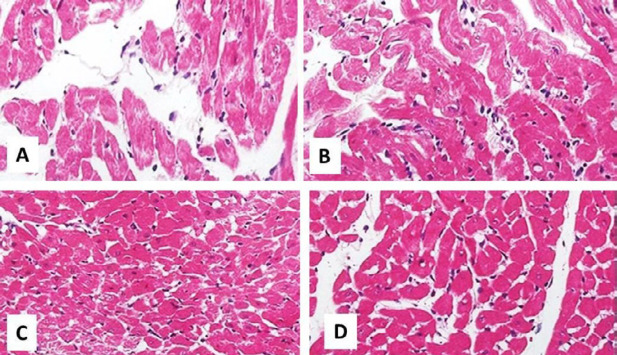
Micrographs of cardiomyocyte demonstrating the process of apoptosis in experimental I/R diabetic hearts. Myocardial apoptosis was identified using TUNEL staining

Apoptosis rate in non-treatment I/R groups was far higher than the other treatment groups. There was a moderate number of apoptotic cells in the I/R+TQ group of control hearts (Figure 4). IPostC or TQ could not decrease the apoptotic cells in diabetic hearts (Figure 5). In a sense, a few apoptotic cells were detected in I/R with IPostC plus TQ groups both in control and diabetic rats (Figures 4 and 5). Furthermore, TQ alone (*P=*0.02) or in combination with IPostC plus TQ (*P*<0.01) significantly decreased AI in control hearts compared with the corresponding I/R group (Figure 3A). The effect of combination treatment was greater than those of single treatments, and only the combination treatment with TQ and IPostC reduced AI in diabetic hearts as compared with diabetic I/R hearts (*P=*0.01). 

**Table 1:** General characteristics: Blood glucose (mmol/l), body weight (g), heart weight (g), and ratio of heart weight to body weight (%) in control and diabetic rats

Groups	Blood glucose (mmol/l)	BW (g)	HW (g)	HW/BW (%)
Controls	6.23 ± 0.51	271 ±12	1.21 ± 0.09	0.44
Diabetics	31.65 ± 6.1	209 ±13*	1.08 ± 0.08	0.51*

N=30/group, values are mean±SEM. T-test was applied for comparison between two groups; *P<0.01 as compared with the control (non-diabetics) group. BW: Bodyweight; HW: Heart weight

## Discussion

The global prevalence of diabetes has grown sharply, which has become one of the most serious health problems universally. Diabetes is correlated with a higher risk of cardiovascular complications including IHD, coronary artery disease (CAD), congestive heart failure (CHF), and acute myocardial infarction (AMI) (21). Hyperglycemia considerably impaired mitochondrial morphology and function, causing electron leakage and O_2_^•−^ generation, which conditions are key contributors to enhanced myocardial vulnerability to IRI (22). Diabetes destroys the intracellular signaling pathways and mediators accountable for resistance against cell death. 

Our results revealed that there are several major findings in the present study. Firstly, TQ could protect the myocardium against reperfusion injury in non-diabetic animals by dramatically enhancing GSK-3β phosphorylation and Bcl-2 activation and whereby decreasing apoptosis and inflammatory responses. Secondly, TQ or IPostC couldn’t significantly protect the myocardium in type 1 diabetes during IRI. Notwithstanding, co-treatment by IPostC plus TQ in the diabetic animals alleviated apoptosis and inflammatory response markedly and had more potent cardioprotective effects.

During reperfusion, blood flow may not return consistently to all portions of the previously ischemic organ; this circumstance has been characterized as the “no-reflow” phenomenon (23). IRI occurs in the first minutes of reperfusion, and therefore this period shows an appreciated “window of chance” for myocardial protection. The most effective approaches, such as IPostC, are performed in the first minutes of reperfusion (24). Indeed, IPostC could restrict the generation of reactive oxygen species (ROS), which was obtained from the mitochondrial electron transport chain during early reperfusion (25). In this regard, *N. Sativa* (known as black cumin seed) is one of the most promising medicinal plants, the extraordinary biological activity of *N. Sativa* is ascribed to its oil component, TQ (26). Several previous studies have demonstrated the cardioprotective effect of Quinone and phenolic compounds on myocardial IRI models (27, 28). Regarding the cardioprotective effects of TQ against IRI, Gonca and Kurt (29) revealed that pre-treatment with TQ reduced arrhythmia scores during the reperfusion period. Lu and colleagues demonstrated that TQ is efficient in decreasing myocardial I/R injury via stimulation of the SIRT1 pathway which can decrease mitochondrial oxidative stress injury and apoptosis (13). Another study revealed that TQ can effectively develop the cardiac function and exert its anti-oxidative and anti-apoptotic activities involving the modulation of autophagy (30). Furthermore, Liu and *et al*. demonstrated that TQ treatment significantly enhanced insulin levels and body weight, and decreased blood glucose and heart rate levels in diabetic animals (31). 

Protecting mitochondria from oxidative injury is a rational treatment method to decrease IRI (13). mPTP has been recognized as a crucial modulator of cardiac IRI. It has been well documented that mPTP remains closed throughout the ischemia, whilst it becomes open in the early minutes of reperfusion. mPTP opening is generally induced through the accumulation of ROS, enhanced cytosolic and mitochondrial matrix Ca_2_^+^ levels, and oxidative stress. Inhibiting the opening of mPTP with its specific blocker including cyclosporin A (CsA) pre/post-ischemia may attenuate myocardial IRI in these patients. Hence, mPTP is a pivotal therapeutic objective for preventing IRI (6, 32). Moreover, apoptosis plays a key role in the pathophysiology of IRI both in diabetic and non-diabetic conditions (33). Apoptosis is divided into two distinct pathways including extrinsic (receptor-dependent) and intrinsic (mitochondria-dependent). Cell apoptosis via the mitochondria pathway is mediated by Bcl-2 family proteins, which is categorized into two groups: anti-apoptotic members, including Bcl-2 and Bcl-xL, and pro-apoptotic members, including Bak, Bad, Bax, and Bid (4). The Bcl-2/Bax balance is vital for sustaining cell homeostasis. mPTP opening may be controlled by Bcl-2 and Bax (32). Bax may mediate mPTP opening through binding of adenine nucleotide translocase (ANT) or voltage-dependent anion channel (VDAC), whilst Bcl-2 may directly impede the interaction between Bax and VDAC/ANT to obstruct the mPTP opening (34). STZ-induced type 1 diabetes resulting in myocardial apoptosis was significantly increased compared with the control group, which is due to cytochrome c release leading to caspase-3 activation and then cell death (10). Our results revealed that STZ-induced chronic diabetes in rats markedly elevated apoptosis (evaluated through comparing TUNEL-positive cells) in IRI in comparison with those of controls. 

Also, elevated levels of ROS during reperfusion can induce inflammatory response and overproduction of pro-inflammatory cytokines following I/R insult (35). Important cytokines like TNF-α, IL-1, and IL-6 are the beginning promoters of the inflammation during IRI (36). TNF-α is mainly produced by macrophages, which is involved in the formation and progression of myocardial IRI and promotes myocardial cell apoptosis, thus increasing myocardial damage (37). Inhibition of inflammatory response is considered a cardioprotective mechanism, which is IPostC reduced reperfusion injury. Herein, we revealed that the administration of TQ in non-diabetic hearts diminished apoptosis and inflammatory cytokines in isolated I/R hearts; besides, co-treatment with TQ plus IPostC had a very potent favorable effect on those parameters in control groups. Furthermore, in diabetic groups, TQ or IPostC alone failed to provide any protection and, in a sense, diabetes abrogated the positive effects of TQ or IPostC on the myocardium. Nevertheless, co-treatment of TQ plus IPostC revealed complete cardioprotection and statically significant diminishing levels of inflammatory cytokines and apoptosis.

Activation of PI3K/Akt signaling is critically involved in protecting the cardiomyocyte against IRI (33). PI3K is recognized as a crucial player in the survival pathway, which has been involved in IPostC protection. Reduction of the amount of phosphorylation and activity of PI3K is one of the causes of diabetes (38). PI3K activation induces phosphorylation of the Ser/Thr kinase Akt which afterward inhibits the formation of Bcl-2 family proteins, stimulates endothelial nitric oxide synthase (eNOS), protein kinase C (PKC), and mTOR/p70s6K, and obstructs downstream GSK-3β (39). GSK-3β is a pro-apoptotic kinase, which is a pivotal player in diabetic cardiomyopathy and myocardial IRI (40). GSK-3β is actuating in normal and non-phosphorylated state and can open the mPTP, contributing to the release of cytochrome c into the cytosol and starting the apoptosis and oxidative reactions (41). On the other hand, mPTP closure is attained by phosphorylation and repression of GSK-3β at Ser 9. IPostC may inhibit the activity of GSK-3β by its phosphorylation, and hence, enhance the cell tolerance to oxidative damage (42). Herein, the phosphorylation of GSK-3β in diabetic rats was markedly fewer than the control group. The suppression of phosphorylation of GSK-3β in diabetic rats was correlated with enhanced cardiac damage and apoptosis as compared with healthy rat hearts. The amount of GSK-3β was the same in diabetic and non-diabetic groups of our study and this indicates that chronic diabetes diminishes the power of IPostC to phosphorylate GSK-3β, keeping this detrimental protein kinase in its active form. Therefore, we hypothesized that type 1 diabetes mellitus might have a negative effect on these crucial pathways involved in IPostC. Indeed, the PI3K/Akt/GSK3β pathway is upstream of mPTP signal transduction, and was not phosphorylated efficiently by IPostC in diabetic groups as compared with the healthy control animals.

## Conclusion

Given together, the results of the present study demonstrate that administration of TQ could exert a cardioprotective effect against reperfusion injury in non-diabetic animals by significantly enhancing GSK3β phosphorylation and Bcl-2 activation, hence, decreasing apoptosis and inflammatory responses. Also, co-administration of TQ with IPostC can protect the diabetic myocardium during IRI by attenuating the apoptosis and inflammatory response. Bearing all this in mind, TQ improves the potency of IPostC on activating the survival protein kinases and mitigation of the mPTP opening, eventually resulting in cardioprotection during IRI induction in diabetic rats.
